# A virus plays a role in partially suppressing plant defenses induced by the viruliferous vectors

**DOI:** 10.1038/s41598-018-27354-9

**Published:** 2018-06-13

**Authors:** Pei Li, Huan Liu, Fei Li, Xiaolan Liao, Shahbaz Ali, Maolin Hou

**Affiliations:** 1 State Key Laboratory for Biology of Plant Diseases and Insect Pests, Institute of Plant Protection, Chinese Academy of Agricultural Sciences, Beijing, 100193 China; 2 College of Plant Protection, Hunan Agricultural University, Changsha, 410128 China; 3 Scientific Observing and Experimental Station of Crop Pests in Guilin, Ministry of Agriculture, Guilin, 541399 China; 4 Southern Regional Collaborative Innovation Center for Grain and Oil Crops in China, Changsha, 410128 China

## Abstract

Herbivorous attack induces plant defenses. There is evidence that some pests suppress these defenses by interfering with signaling pathways. We here report that infestation by the white-backed planthopper, *Sogatella furcifera*, induces defense responses in rice and infection of the southern rice black-streaked dwarf virus in the planthoppers partially suppresses the planthopper-induced plant defenses. Salicylic acid (SA) levels generally showed a temporal increase pattern while jasmonic acid (JA) levels generally exhibited a decrease pattern in the planthopper-infested plants, irrespective of virus infection status in the insects. The increase in SA was less while the decrease in JA was more in the viruliferous insect-infested plants than in the nonviruliferous insect-infested plants at both 48 and 72 h post infestation. The phytohormone levels corresponded to the patterns of relative expression levels of SA-marker genes (*ICS1* and *NPR1*) and JA-marker gene (*AOS2*) in the plant treatments. Planthoppers performed better on the uninfested plants than on the previously infested plants and were of not significant increase in performance on the plants previously attacked by viruliferous planthoppers in comparison with the plants previously attacked by nonviruliferous insects. Our results indicate that the virus plays a role in partially suppressing the plant defenses induced by the planthopper. These findings provide a new perspective on plant–virus-vector interactions.

## Introduction

Most of the plant viruses rely on insects for spread^1^. Complex interplay has evolved in the triangle relationship among virus, plant and insect vector. The direct (by infection of the vector) or indirect (by infection of the host plant) interaction between plant virus and vector can be beneficial, neutral, or deleterious for the vector^2–4^.

The southern rice black-streaked dwarf virus (SRBSDV) is a *Fijivirus* transmitted in a persistent propagative manner^5^. In recent years, SRBSDV have devastated rice crops in south China and Vietnam and caused large economic losses^6,7^, and occurrence was also reported in Japan^8^. The white-backed planthopper (WBPH), *Sogatella furcifera* Horváh, is the only known vector of SRBSDV^7^. The latent periods of SRBSDV in WBPH varies from 6 to 14 d^9^. Viruliferous WBPH nymphs experience extended development than nonviruliferous nymphs and nonviruliferous WBPH adults live longer when they are fed on SRBSDV-infected plants, which are believed to favor virus spread^4^.

When attacked by herbivores or pathogens, plants usually mobilize an array of defensive responses to counteract the attack, which are primed by phytohormones such as salicylic acid (SA), jasmonic acid (JA) and ethylene (ET)^10^. Biotrophic pathogens, virus included, and most phloem-feeding insects may induce SA pathway, while necrotrophic pathogens including some viruses, some chewing herbivores, and some phloem-feeding insects may induce JA response^11–14^.

A number of functional genes have been identified for the biosynthesis of phytohormones in priming of the induced defense responses. For example, *NPR1* genes are first reported in SA-mediated systemic acquired resistance in *Arabidopsis*^15^. *ICS1* genes are required for pathogen-induced biosynthesis of salicylic acid^16^. *AOS* and *LOX* genes are involved in JA biosynthesis^17^. In rice plants damaged by the brown planthopper *Nilaparvata lugens*, SA level is increased and SA pathway genes are up-regulated, but there are no significant changes in JA level and expression of JA pathway genes in comparison with those in the undamaged plants^18^. In SRBSDV-infected rice plants, the expression of JA and SA biosynthesis genes changed dynamically and the JA gene *OsAOS1* was down-regulated at 40 days post inoculation while the SA gene *OsICS* was up-regulated at 35 days post inoculation when the viral titers were the highest^19^. The induced defense responses triggered by a previous attack by the spider mite *Tetranychus evansi* and *T. urticae* are modulated by JA-related genes and show influence on the performance of subsequent *T. evansi* infestation^20^. However, in the SRBSDV-rice-WBPH system, it remains unknown whether virus infection of WBPH would affect plant defenses induced by a previous attack and what is the consequence for subsequent conspecific infestation.

Proteinase inhibitor (PI) is an inducible defense-related protein that is regulated by JA signal pathway^21,22^. PI is known to inhibit activities of digestive enzymes in insects’ midguts, thus reducing the growth and development of insects^23^. The spider mite *T. evansi* performed better on the previously conspecifics-infested plants due to these plants having lower PI activity^20^, PI activity in tomato leaves infested by tomato yellow leaf curl virus (TYLCV)-infected *Bemisia tabaci* biotype Q was lower than in leaves infested by nonviruliferous insects^3^. Whether previous infestation by viruliferous vector would influence on plant PI activity differentially and further affect conspecifics performance is unclear.

In this study, we measured JA and SA concentrations and PI activity, and quantified JA- and SA-related gene expression in healthy plants and plants previously exposed to heavy infestation of viruliferous or nonviruliferous WBPH. Further, we compared the performance of nonviruliferous WBPH on these plants. Our goals are to understand how induced plant defenses would affect conspecifics performance and to determine whether virus infection of the planthopper will affect the induced plant defense responses.

## Results

### Plant endogenous SA and JA concentrations

**Figure 1:**
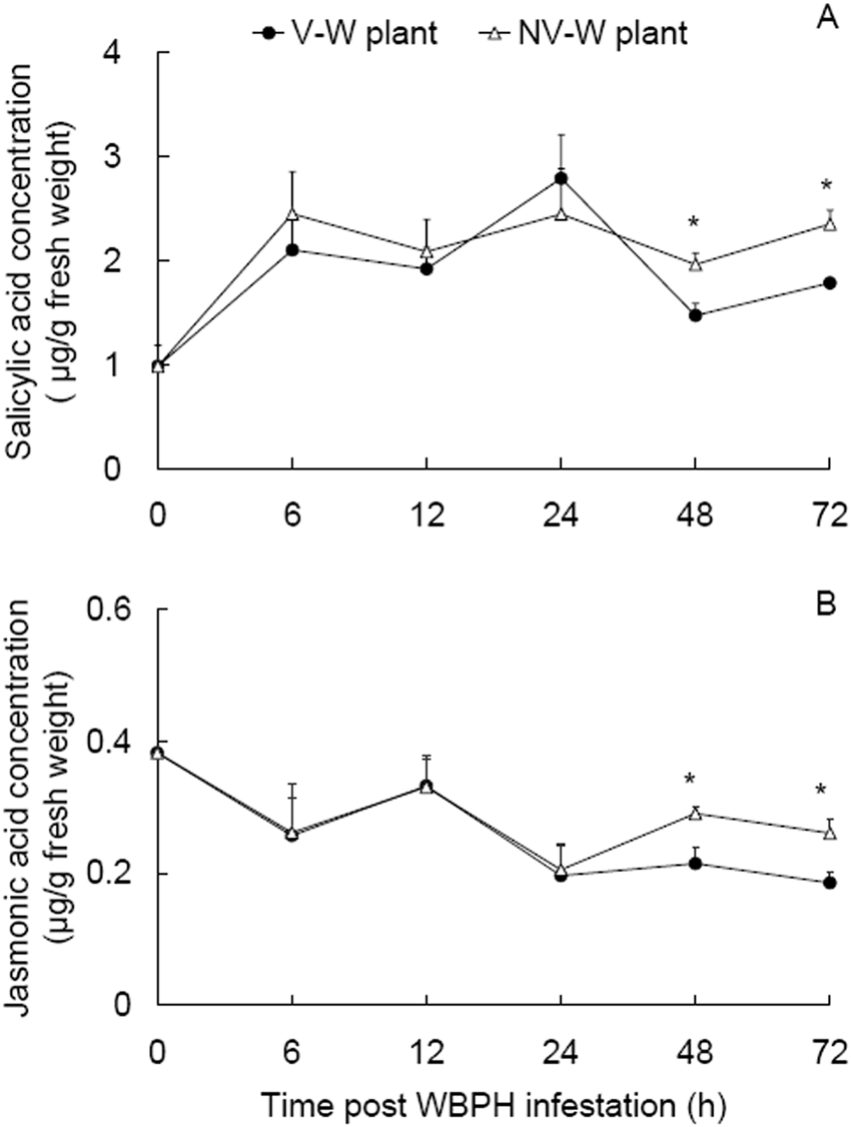
Dynamic concentrations of salicylic acid (**A**) and jasmonic acid (**B**) in viruliferous and nonviruliferous WBPH-infested plants (V-W and NV-W plant, respectively) and uninfested plants (0 h post WBPH infestation). Values are means ± SE. * indicates significant differences between the viruliferous and nonviruliferous WBPH-infested plants at a certain time point post WBPH infestation (independent sample *t*-test, *P* < 0.05).

Endogenous SA and JA concentrations in the infested rice plants were measured dynamically (Fig. 1). SA concentrations in the WBPH-infested plants generally showed a temporal increase pattern post WBPH infestation (Fig. 1A). Between the plants infested by viruliferous and nonviruliferous WBPH, SA concentrations showed no significant differences at 6, 12 and 24 h post infestation (hpi) (*t* ≤ 0.659, *P* ≥ 0.534), but were 33.3% and 31.7% lower at 48 and 72 hpi in the viruliferous WBPH-infested plants than in the nonviruliferous WBPH-infested plants (*t* ≥ 3.088, *P* ≤ 0.021), respectively (Fig. 1A).

Unlike SA, JA levels in the WBPH-infested plants generally exhibited a temporal decrease pattern post WBPH infestation (Fig. 1B). JA levels were not different between the viruliferous and nonviruliferous WBPH-infested plants at 6, 12 and 24 hpi (*t* ≤ 0.144, *P* ≥ 0.89), but were 35.3% and 40.7% lower at 48 and 72 hpi in the former than in the latter (*t* ≥ 3.131, *P* ≤ 0.033), respectively (Fig. 1B).

### Relative expression levels of SA and JA genes

**Figure 2:**
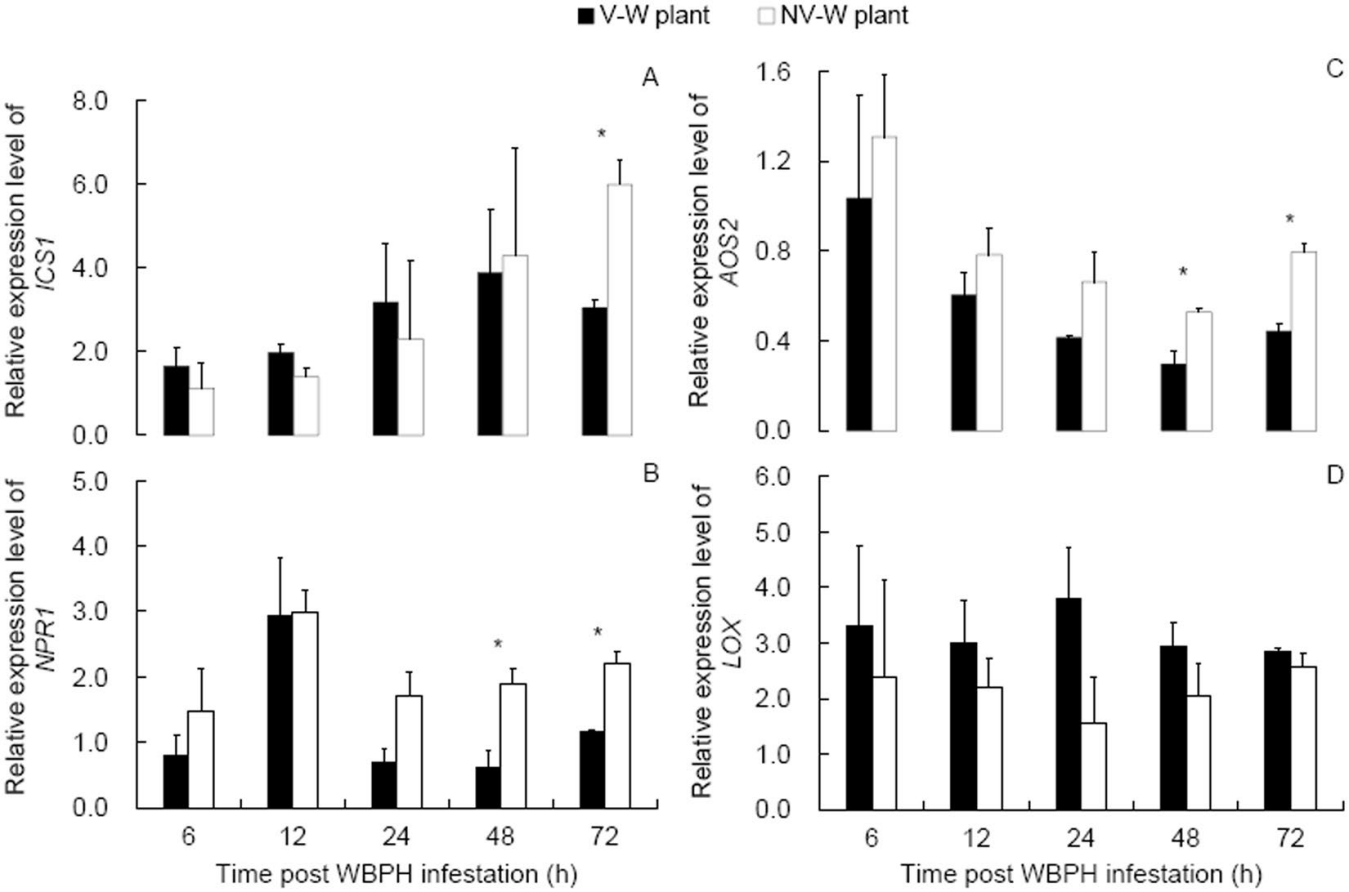
Dynamic expression levels of salicylic acid-marker genes (A: *ICS1*, B: *NPR1*) and jasmonic acid-marker genes (C: *AOS2*, D: *LOX*) in the viruliferous and nonviruliferous WBPH-infested plants (V-W plant and NV-W plant, respectively) relative to those in the uninfested plants. Values are means ± SE. * indicates significant differences between the viruliferous and nonviruliferous WBPH-infested plants at a certain time point post WBPH infestation (Independent sample *t*-test, *P* < 0.05).

Expression levels of the SA-marker genes *ICS1* and *NPR1* and the JA-marker gene *AOS2* all showed significant changes with the time post WBPH infestation of the plants (*ICS1*: *F* = 3.624, df = 5,30, *P* = 0.011; Fig. 2A; *NPR1*: *F* = 5.671, df = 5,30, *P* = 0.001; Fig. 2B; *AOS2*: *F* = 3.770_,_ df = 5,30, *P* = 0.009; Fig. 2C), while the JA-marker gene *LOX* did not (*F* = 1.483, df = 5,30, *P* = 0.225; Fig. 2D). In the SA pathway, *ICS1* was expressed at a lower level in the plants infested by viruliferous than nonviruliferous WBPH at 72 hpi (t = 4.867, *P* = 0.008; Fig. 2A), and *NPR1* showed similar patterns at both 48 and 72 hpi (*t* ≥ 3.835, *P* ≤ 0.024; Fig. 2B). For *AOS2*, expression was at lower levels at both 48 and 72 hpi in the viruliferous than in the nonviruliferous WBPH-infested plans (*t* ≥ 3.657, *P* ≤ 0.022; Fig. 2C). The JA-marker gene *LOX* showed no significant differences in expression level between the viruliferous and nonviruliferous WBPH-infested plants at all the time points post WBPH infestation (*t* ≤ 1.802, *P* ≥ 0.146; Fig. 2D).

### Proteinase inhibitor activity

**Figure 3:**
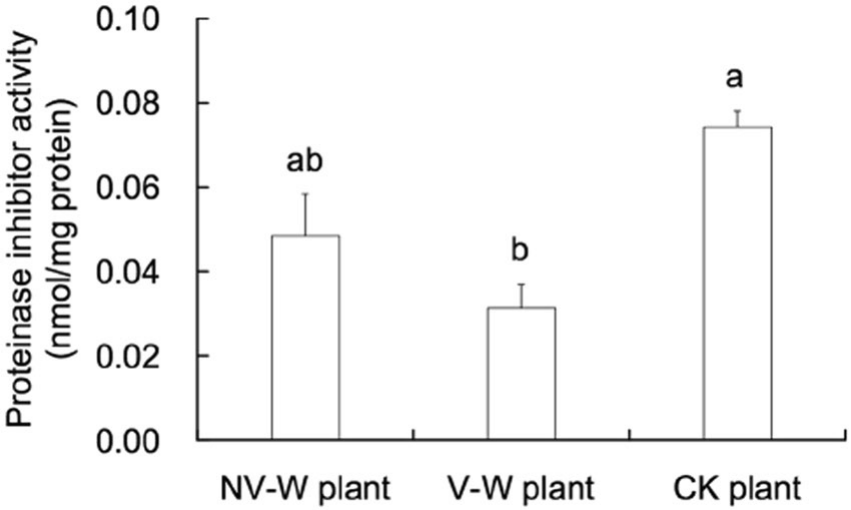
Proteinase inhibitor activity in the viruliferous and nonviruliferous WBPH-infested plants (NV-W plant and V-W plant, respectively) and uninfested plants (CK plant). Values are means ± SE. Different letters over the bars indicate significant differences (Tukey HSD test, *P* < 0.05).

Proteinase inhibitor activity was reduced in the infested plants (*F* = 9.956, df = 2,14, *P* = 0.003; Fig. 3). It was significantly lower in the viruliferous WBPH-infested plants than in the uninfested plants (Tukey HSD test, *P* = 0.002), but was not significantly different between the viruliferous and nonviruliferous WBPH-infested plants.

### WBPH performance

**Figure 4:**
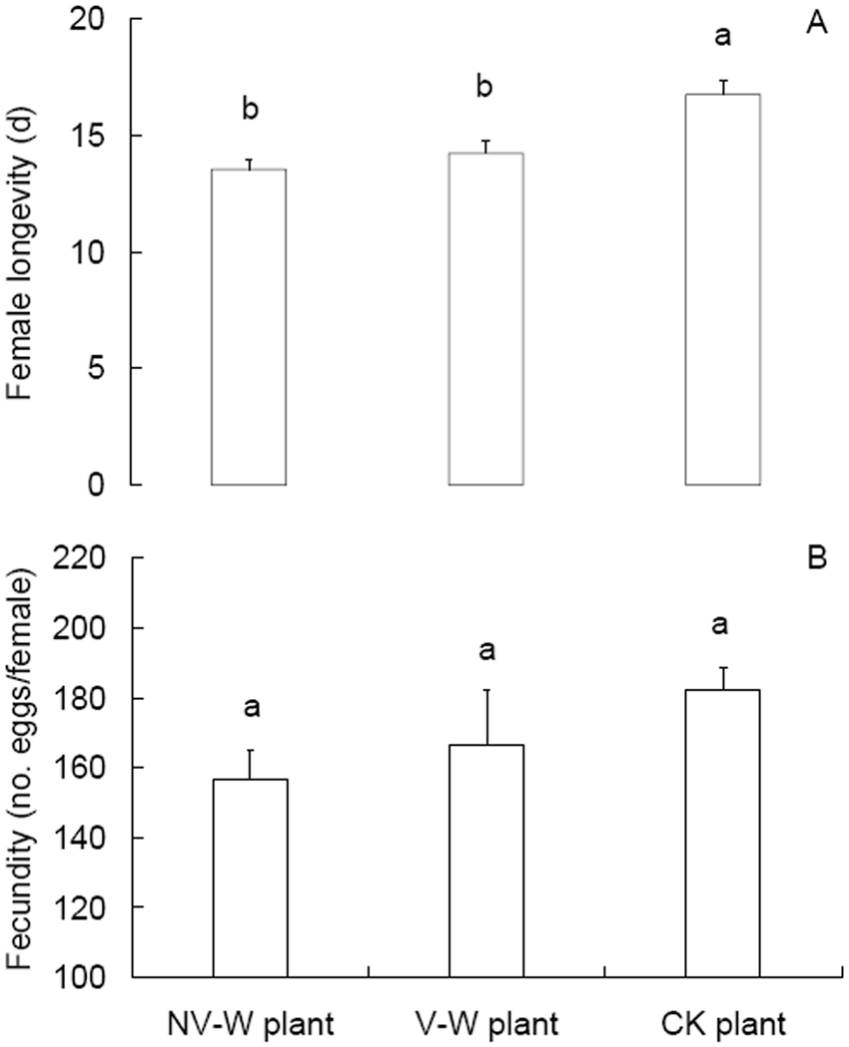
Longevity (**A**) and fecundity (**B**) of nonviruliferous WBPH females feeding on the rice plants previously infested or not. NV-W plant: plants previously infested by nonviruliferous females; V-W plant: plants previously infested by viruliferous females; CK plant: plants previously not infested. Values are means ± SE. Different letters over the bars indicate significant differences (Tukey HSD test, *P* < 0.05).

When the plants previously infested by WBPH or not were subsequently subjected to nonviruliferous WBPH females, significant difference was observed in the insects’ longevity (*F* = 9.023, df = 2,74, *P* < 0.001; Fig. 4A). The females lived shorter on the previously WBPH-infested plants than on the uninfested plants (Tukey honestly significant difference (HSD) test, *P* ≤ 0.008); on the infested plants, the females lived 14.2 d on the viruliferous WBPH-infested plants and 13.6 d on the non-viruliferous WBPH-infested plants; however, the difference was not significant. Fecundity of the females was not significantly different between the plant treatments (*F* = 1.44, df = 2,60, *P* = 0.245; Fig. 4B), although was reduced by 15.8% on the infested plants in comparison with the uninfested plants and by 5.8% on the non-viruliferous WBPH-infested plants compared to the viruliferous WBPH-infested plants.

## Discussion

We found that the WBPH on the plants previously infested by the conspecifics did not perform as well as those on the uninfested plants, as indicated by shorter longevity and a not significant reduction (by 15.8%) of fecundity. This corresponds to what is normally observed as the effect of induced plant defense, i.e., herbivore performance is lower on previously damaged plants than on undamaged plants^24^. However, this result contrasts to the findings by Sarmento *et al*.^20^, where the spider mite *T. evansi* performed much better on tomato leaves previously attacked by the conspecifics than on unattacked leaves while had reduced performance on leaves previously attacked by its congener *T. urticae* in comparison with uninfested plants. The recorded patterns of higher performance of *T. evansi* on the plants coincided with these plants having lower PI activity^20^. The low PI activity in leaves previously attacked by *T. evansi* is due to lack of up-regulation of the JA and SA defensive pathways^20^. In our current study, PI activity was lower in the viruliferous WBPH-infested plants or not significantly reduced in the nonviruliferous WBPH-infested plants than that in the undamaged plants (Fig. 3), showing that the insect performance was negatively connected with PI activity, as reported for the brown planthopper *N. lugens*^25^. Therefore, in contrast to the results for *T. evansi*^20^, our results show no positive connection between WBPH performance and PI activity in the previously damaged and undamaged plants.

Plant SA concentrations showed a general temporal increase pattern within 72 h of previous infestation by WBPH (Fig. 1A). Although SA levels and expression of SA-related genes may show circadian rhythm, as that reported for the expression of the serine/threonine protein kinase gene *OsPBL1*^26^, Silverman *et al*.^27^ reported no significant changes in SA levels in healthy rice seedlings during a five week monitoring (7–35 days after sowing). Even with circadian rhythm, the SA levels and SA-related gene expression may show similar circadian changes in different treatments in the present study, as in the case of *OsPBL1*^26^. Therefore, although not measured dynamically in the uninfested plants, it can be reasoned that SA levels in the uninfested plants may be low in comparison with those in the infested plants. The general temporal increase pattern of SA concentrations in the infested plants (Fig. 1A) is linked with the low WBPH performance on these plants (Fig. 4). In the brown planthopper *N. lugens*, the insect performance was positively correlated with planthopper-induced H_2_O_2_ and SA concentrations^25^. In another study, WBPH showed no performance difference on the rice mutants with impaired JA biosynthesis and the wild lines^28^. Our current results and previous reports indicate that the up-regulated SA signaling pathway may explain the relatively poor performance of WBPH on the previously infested plants in comparison with that on the uninfested plants.

Between the viruliferous and nonviruliferous WBPH-infested plants, we observed a not significant reduction in conspecifics performance on the latter in comparison with the former (Fig. 4), which corresponds to the higher SA concentrations in the latter than in the former at 48 and 72 hpi (Fig. 1). In the interaction between *B. tabaci* and TYLCV, *B. tabaci* biotype Q has a mutualistic relationship with TYLCV in that viruliferous biotype Q down-regulated while viruliferous biotype B up-regulated SA signaling, which is believed to be the reason for the wide spread of *B. tabaci* biotype Q and TYLCV in China^3^. Our results indicate that SRBSDV infection of WBPH functions in a way to down-regulate the induced SA-related plant defenses.

Plant defense responses to sucking insect pests are principally regulated by SA pathways and may act on subsequent infestation by conspecifics or congeners^29,30^. Attack by phloem-feeding insects usually induce SA accumulation^25,31^, as observed in our results (Fig. 1A). The temporally increased SA concentration in the infested plants coincides with the up-regulation of the SA-marker genes *NPR1* and *ICS1* in these plants (Fig. 2A,B). These results confirm previous reports that planthopper infestation induces the SA pathway^25,28^. Interestingly, the recorded lower SA concentration in the viruliferous than in the nonviruliferous WBPH-infested plants corresponds to the down-regulation of the SA-marker genes *NPR1* and *ICS1* in the former than in the latter (Figs 1A and 2A,B). In contrast to our results, the SA gene *OsICS* was up-regulated in SRBSDV-infected rice plants when the viral titers were the highest at 35 days after virus inoculation in comparison with that in uninfected plants^19^. However, this result^19^ has to be taken as the plant defense response to SRBSDV alone; while in the present study, the SA levels were measured within 3 days of infestation by the planthoppers and the plants may have responded to both virus infection and WBPH infestation. In a pathosystem consisting of a DNA virus TYLCV, *B. tabaci* and tomato plants, different patterns were reported. Infestation by viruliferous *B. tabaci* biotype B increased plant SA levels and up-regulated SA genes (*NPR1* and *PR1*) in comparison with infestation by the nonviruliferous counterparts while infestation by viruliferous *B. tabaci* biotype Q showed no influence on SA levels and expression of SA genes (*NPR1* and *PR1*)^3^. These results indicate that the responses of plant SA signal pathway to the infestation of viruliferous vectors may vary with the specific pathosystems in case.

We recorded temporally reduced JA concentrations in the infested plants (Fig. 1). Although not measured dynamically, as reasoned for SA levels, JA levels in the infested plants may be low in comparison with those in the uninfested plants, which coincides with the down-regulation of the JA-marker gene *AOS2* in the infested plants (Fig. 2C), while the gene *LOX* is up-regulated in the infested plants. Previous reports showed similar JA marker gene expression patterns in rice plants after bacterial blight disease inoculation, i.e., up-regulated *LOX* and reduced *AOS2*^31^ and in tomato plants infested by *B. tabaci*, i.e. up-regulated JA upstream gene *LOX* and reduced downstream gene *PI II* and JA concentrations^32^.

In conclusion, our results demonstrate that previous WBPH infestation increases the SA-mediated plant defenses, which accounts for the reduced performance of subsequent WBPH infestation on the infested vs uninfested rice plants. Compared to previous infestation by nonviruliferous WBPH, previous infestation by viruliferous insects up-regulates SA to a lesser extent, which contributes to the not significantly increased WBPH performance on the plants previously infested by viruliferous WBPH over the plants infested by nonviruliferous insects. The results show that infection with SRBSDV in WBPH plays a role in partially suppressing the plant defenses induced by the vector, which provides a new perspective on plant–virus-vector interactions and additional information for assessing SRBSDV transmission risks and field epidemiology.

## Methods

### Insects and plants

Potted seedlings of a SRBSDV-susceptible rice variety (Diantun 502) were cultured within 80-mesh insect-proof cages (50 by 50 by 50 cm) in a greenhouse (30 ± 5°, 15 L: 9D). WBPH colonies were maintained using caged rice seedlings in a climatic chamber (30 ± 1°, 15 L: 9D). SRBSDV-positive seedlings collected from paddy fields, as determined by reverse transcription polymerase chain reaction (RT-PCR), were used to establish a stock culture of infected rice plants in cages within another climatic chamber.

To obtain viruliferous WBPH adults for the experiments, nonviruliferous young nymphs (1st to 2nd instars) of WBPH were confined with SRBSDV-positive plants for 5 d and then transferred to caged virus-free plants for development. Newly emerged adult insects (<24 h) were used in assays.

### SRBSDV detection by RT-PCR

Virus infection status was detected by one-step RT-PCR as described by Li *et al*.^33^. Briefly, total RNA of each sample was amplified using primers (forward: 5′-CGCGTCATCTCAAACTACAG-3′, reverse: 5′-TTTGTCAGCATCTAAAGCGC-3′)^34^. The amplified fragment of the expected size (682 bp) of SRBSDV-S10 fragment was confirmed by electrophoresis in agarose gels. An insect designated as viruliferous was confirmed by RT-PCR as SRBSDV-positive after the experiment with the insect was finished, and a plant designated as infected was confirmed as SRBSDV-positive using a portion of the plant material. In our laboratory colonies, about 80% of the insects and plants designated as SRBSDV-positive were confirmed to be really SRBSDV-positive.

### Sampling for determination of defense-related phytohormone pathways and proteinase inhibitor activity

To determine the effects of WBPH infestation and SRBSDV infection of the WBPH on defense related phytohormone pathways and proteinase inhibitor activity, one 35–45 day old rice seedling was exposed to 20 viruliferous or nonviruliferous macropterous females in a plastic tube (3 cm × 8 cm) within a climatic chamber (27 ± 2 °C, 15 L: 9D, RH 75%). A sponge disc (3 cm in diameter and 2 cm thick) was used to secure the seedling at 6 cm above roots and another sponge disc was used to seal the tube opening, thus leaving a space of 4-cm height in the tube, where the insects were left to feed ad lib. Leaf sheaths of the 4-cm stem segments were sampled at 0 (not infested), 6, 12, 24, 48, or 72 h post infestation (hpi) by WBPH and frozen in liquid nitrogen. The leaf sheath samples thus collected were used to measure the concentrations of salicylic acid and jasmonic acid and to determine the relative expression levels of phytohormones-related genes. Additionally, the 72 hpi samples were used to determine proteinase inhibitor activity.

### Quantification of phytohormone concentrations

Phytohormones were quantified by liquid chromatography-mass spectrometry to detect the influence of WBPH infestation of the plants and SRBSDV infection of the WBPH on phytohormone levels in rice plants. Total phytohormones were extracted and purified as described by Kojima *et al*.^35^. Briefly, radio-labeled internal standard containing 50 ng D_6_-SA (Sigma, cat no. 616796) and 50 ng H_2_-JA (OIChemim, cat no. 0145324) was added to the sample during the extraction^36^. The extracted sample was transferred by pipette to a brown glass vial and then analyzed using a triple quadruple liquid chromatography-mass spectrometry system (XEVO TQ-S-Quantum Access, Waters, USA). The reaction monitoring conditions and gradient parameters were listed in the supplemental file (Tables S1 and S2). The hormone concentration was normalized as ng per g of fresh weight of leaf sheath using the mass of fresh plant tissue measured before extraction. The quantification was repeated from four to eight samples and for each sample, technically repeated for three times.

### Analysis of relative expression of phytohormones-related genes

To measure the impact of WBPH infestation of the plants and SRBSDV infection of the WBPH on induced phytohormone response in rice plants, we quantified transcript levels of SA- and JA-related genes in rice leaf sheath using quantitative real-time PCR (qPCR). *ICS1* (isochorismate synthase 1) and *NPR1* (homolog of *Arabidopsis* nonexpressor of pathogenesis-related genes 1) were selected as SA-marker genes, and *LOX* (lipoxygenase) and *AOS2* (allene oxide synthase 2) were selected as JA-marker genes (Table 1). The extraction of total RNA from the leaf sheath samples and the synthesis of cDNA was described by Li *et al*.^37^. Every attempt was made to adhere to minimum information for quantitative real-time PCR experiments (MIQE) guidelines to ensure proper and accurate reporting of qPCR data^38^. The qPCR reaction was performed using the Bester SybrGreen qPCR mastermix (DBI Bioscience, Germany) with the 7500 Sequence Detection System (Applied Biosystems, Foster City, CA, USA). Amplification reactions were performed in a 20 μL final volume containing 10 μL of Bester SybrGreen qPCR mastermix (DBI), 0.4 μL of forward primer (10 μM) and reverse primer (10 μM) pairs (Table 1), 0.04 μL of 50 × Rox, and 5 μL of cDNA (4 ng/μL) and 5.16 μL of sterilized H_2_O. Reaction conditions were as follows: 95 °C for 2 min followed by 40 cycles of 10 sec at 95 °C, 34 sec at 60 °C, and then followed by melt curves stages. Negative controls without template were included in each experiment. The qPCR reaction was performed for three samples and for each sample, technically repeated for three times. The comparative 2^−ΔΔCT^ method was used to calculate the relative gene expression levels in different samples^39^. The average of CT values was used to calculate ΔΔCTs with the following equation:

$$\begin{array}{c}{\rm{\Delta }}{\rm{\Delta }}\mathrm{CT}=({\rm{Average}}\,{{\rm{CT}}}_{{\rm{Target}}{\rm{gene}}}-{\rm{Average}}\,{{\rm{CT}}}_{{\rm{Reference}}{\rm{gene}}}){\rm{of}}\,{\rm{infested}}\,{\rm{plantsamples}}\\ \,\,\,\,\,-({\rm{Average}}\,{{\rm{CT}}}_{{\rm{Target}}{\rm{gene}}}-{\rm{Average}}\,{{\rm{CT}}}_{{\rm{Reference}}{\rm{gene}}}){\rm{of}}\,{\rm{uninfested}}\,{\rm{plantsamples}}.\end{array}$$

**Table 1:** Nucleotide sequence of primers used for qPCR analysis.

Gene name	GenBank No.	Sequence (5′-3′)	Expected length (bp)	Reference
Target gene				
*ICS1*	AK120689	TATGGTGCTATCCGCTTCGAT	120	Qiu *et al*.^31^
		CGAGAACCGAGCTCTCTTCAA		
*NPR1*	AY923983	TTTCCGATGGAGGCAAGAG	120	Chern *et al*.^43^
		GCTGTCATCCGAGCTAAGTGTT		
*LOX*	D14000	GCATCCCCAACAGCACATC	110	Qiu *et al*.^31^
		AATAAAGATTTGGGAGTGACATA		
*AOS2*	AY062258	CTCGTCGGAAGGCTGTTGCT	120	Qiu *et al*.^31^
		ACGATTGACGGCGGAGGTT		
Reference gene				
*UBQ5*	AK061988	AACCACTTCGACCGCCACT	120	Li *et al*.^44^
		GTTCGATTTCCTCCTCCTTCC		
*OsActin*	AB047313	CAGCACATTCCAGCAGAT	108	Hao *et al*.^45^
		GGCTTAGCATTCTTGGGT		

### Determination of proteinase inhibitor activity

A leaf sheath sample was ground at 4 °C using a TissueLyser, and proteinase inhibitor was extracted as described by Sarmento *et al*.^20^. The proteinase inhibitor activity was represented by trypsin activity that was detected at 410 nm with a spectrophotometer as the difference between the absorbance monitored at 150 s and 60 s^40^. The trypsin activity was expressed as mg of trypsin inhibited per g of protein^41^. The measurement was performed for five samples and for each sample, technically repeated for three times.

### Influence of previous WBPH infestation on subsequent conspecifics performance

To measure the performance of WBPH females on plants previously exposed to WBPH of different virus infection status, 35–45 day old potted rice plants were individually exposed to 100 newly emerged viruliferous or nonviruliferous WBPH for 72 h in an insect-proof cage in a completely randomized design. Control plants not exposed to WBPH were also placed in cages. After 72 h, the insects in the cages were removed and the plants were each transplanted into a glass tube (2.5 cm in diameter and 15 cm in length) with nutrient solution^42^. Then one nonviruliferous WBPH female and two males (1 day old) were confined with one plant either previously infested by nonviruliferous or viruliferous WBPH or left uninfested in the tubes in a completely randomized design. The glass tubes were observed daily and nymphs, if any, were removed after their number was recorded. Upon the death of females, leaf sheaths of the rice seedlings were dissected under a stereomicroscope and the number of unhatched WBPH eggs therein was recorded. Female longevity was calculated using dates of emergence and death. Fecundity was calculated as the sum of nymph numbers and number of the unhatched eggs. For each treatment, the bioassays for longevity and fecundity were repeated 15–34 times.

### Statistical Analysis

One-way analysis of variance (ANOVA) was used to detect differences of WBPH performance, relative expression of the genes and PI activity between the plant treatments, i.e., uninfested, nonviruliferous and viruliferous WBPH-infested plants. Tukey HSD test was used to separate the means where there was a significant effects on WBPH performance and PI activity. Differences in JA/SA concentration and relative expression of the genes between nonviruliferous and viruliferous WBPH-infested plants at a specific time post WBPH infestation were compared using independent sample *t-*test (SPSS version 19.0, SPSS Inc., Chicago, IL).

## Electronic supplementary material

Supplemental file
