# Supplementary material for: A virus plays a role in partially suppressing plant defenses induced by the viruliferous vectors
Source: Sci Rep. 2018 Jun 13;8:9027. doi: 10.1038/s41598-018-27354-9 (PMC5997988; doi:10.1038/s41598-018-27354-9)
Supplement: Supplementary file 1 — Supplemental file [file 41598_2018_27354_MOESM1_ESM.doc]

**A virus plays a role in partially suppressing plant defenses induced by its planthopper vector**

Pei Li^1,2,3^, Huan Liu^1,3^, Fei Li^1,3^, Xiaolan Liao^2^, Shahbaz Ali^1,3^ & Maolin Hou^1,3,4^

^1^ State Key Laboratory for Biology of Plant Diseases and Insect Pests, Institute of Plant Protection, Chinese Academy of Agricultural Sciences, Beijing 100193, China; ^2^ College of Plant Protection, Hunan Agricultural University, Changsha 410128, China; ^3^ Scientific Observing and Experimental Station of Crop Pests in Guilin, Ministry of Agriculture, Guilin 541399, China; ^4^ Southern Regional Collaborative Innovation Center for Grain and Oil Crops in China, Changsha 410128, China

| **PH** | **PI** | **DI** | **CV** | **CE** | **IS** | **PI** | **DI** | **CV** | **CE** |
| --- | --- | --- | --- | --- | --- | --- | --- | --- | --- |
| SA | 137.06 | 75.02 | 50 | 28 | d_6_-SA | 144.11 | 69.11 | 52 | 26 |
|  |  | 65.01 | 50 | 24 |  |  | 77.7 | 52 | 24 |
| JA | 209.09 | 59.02 | 60 | 12 | H_2_JA | 211.17 | 59.02 | 48 | 12 |
|  |  | 80.08 | 60 | 24 |  |  | 111.04 | 48 | 22 |

**Table S1** Selected reaction monitoring conditions for protonated or deprotonated plant hormones ( [M−H]−). PH, plant hormones; PI, parents ion; DI, daughters ion；CV, cone voltage; CE, collision energy; IS, internal standard; SA, salicylic acid; JA, jasmonic acid

| **Time (min)** | **Flow** | **A% (H_2_0)** | **B% (Methanol)** | **Curve** |
| --- | --- | --- | --- | --- |
| Initial | 0.3 | 15 | 85 | 6 |
| 1.5 | 0.3 | 85 | 15 | 6 |
| 2.5 | 0.3 | 85 | 15 | 6 |
| 2.6 | 0.3 | 15 | 85 | 6 |
| 5 | 0.3 | 15 | 85 | 6 |

**Table S2** Gradient parameters. Mobile phase A: distilled water with 0.1% formic acid; mobile phase B: methanol.
